# Relationships Among Circulating Levels of Hemostasis Inhibitors, Chemokines, Adhesion Molecules, and MRI Characteristics in Multiple Sclerosis

**DOI:** 10.3389/fneur.2020.553616

**Published:** 2020-10-15

**Authors:** Nicole Ziliotto, Robert Zivadinov, Dejan Jakimovski, Marcello Baroni, Niels Bergsland, Deepa P. Ramasamy, Bianca Weinstock-Guttman, Murali Ramanathan, Giovanna Marchetti, Francesco Bernardi

**Affiliations:** ^1^Department of Life Sciences and Biotechnology, University of Ferrara, Ferrara, Italy; ^2^Department of Neurology, Buffalo Neuroimaging Analysis Center, State University of New York, Buffalo, NY, United States; ^3^Center for Biomedical Imaging at the Clinical Translational Science Institute, State University of New York, Buffalo, NY, United States; ^4^Istituto di Ricovero e Cura a Carattere Scientifico (IRCCS), Fondazione Don Carlo Gnocchi, Milan, Italy; ^5^Department of Pharmaceutical Sciences, State University of New York, Buffalo, NY, United States; ^6^Department of Biomedical and Specialty Surgical Sciences, University of Ferrara, Ferrara, Italy

**Keywords:** multiple sclerosis, neurodegeneration, cerebral microbleeds, hemostasis inhibitors, adhesion molecules

## Abstract

**Background:** Several studies suggested cross talk among components of hemostasis, inflammation, and immunity pathways in the pathogenesis, neurodegeneration, and occurrence of cerebral microbleeds (CMBs) in multiple sclerosis (MS).

**Objectives:** This study aimed to evaluate the combined contribution of the hemostasis inhibitor protein C (PC) and chemokine C-C motif ligand 18 (CCL18) levels to brain atrophy in MS and to identify disease-relevant correlations among circulating levels of hemostasis inhibitors, chemokines, and adhesion molecules, particularly in CMB occurrence in MS.

**Methods:** Plasma levels of hemostasis inhibitors (ADAMTS13, PC, and PAI1), CCL18, and soluble adhesion molecules (sNCAM, sICAM1, sVCAM1, and sVAP1) were evaluated by multiplex in 138 MS patients [85 relapsing-remitting (RR-MS) and 53 progressive (P-MS)] and 42 healthy individuals (HI) who underwent 3-T MRI exams. Association of protein levels with MRI outcomes was performed by regression analysis. Correlations among protein levels were assessed by partial correlation and Pearson's correlation.

**Results:** In all patients, regression analysis showed that higher PC levels were associated with lower brain volumes, including the brain parenchyma (*p* = 0.002), gray matter (*p* < 0.001), cortex (*p* = 0.001), deep gray matter (*p* = 0.001), and thalamus (*p* = 0.001). These associations were detectable in RR-MS but not in P-MS patients. Higher CCL18 levels were associated with higher T2-lesion volumes in all MS patients (*p* = 0.03) and in the P-MS (*p* = 0.003). In the P-MS, higher CCL18 levels were also associated with lower volumes of the gray matter (*p* = 0.024), cortex (*p* = 0.043), deep gray matter (*p* = 0.029), and thalamus (*p* = 0.022). PC-CCL18 and CCL18-PAI1 levels were positively correlated in both MS and HI, PC–sVAP1 and PAI1–sVCAM1 only in MS, and PC–sICAM1 and PC–sNCAM only in HI. In MS patients with CMBs (*n* = 12), CCL18–PAI1 and PAI1–sVCAM1 levels were better correlated than those in MS patients without CMBs, and a novel ADAMTS13–sVAP1 level correlation (*r* = 0.78, *p* = 0.003) was observed.

**Conclusions:** Differences between clinical phenotype groups in association of PC and CCL18 circulating levels with MRI outcomes might be related to different aspects of neurodegeneration. Disease-related pathway dysregulation is supported by several protein level correlation differences between MS patients and HI. The integrated analysis of plasma proteins and MRI measures provide evidence for new relationships among hemostasis, inflammation, and immunity pathways, relevant for MS and for the occurrence of CMBs.

## Introduction

In multiple sclerosis (MS) pathogenesis, blood–brain barrier (BBB) disruption and vascular changes interact in a vicious cycle with altered immune trafficking and the inflammatory processes, supported by adhesion molecules and chemokines (1–3). Several studies also suggested the cross talk of immunity and inflammation with hemostasis, potentially reflected in MS pathogenesis and progression of neurodegeneration (4).

Plasma levels of protein C (PC) have been associated with neurodegenerative magnetic resonance imaging (MRI) outcomes in MS patients (5). Among hemostasis components, PC has coagulation inhibitor activity and also anti-inflammatory and cell protective properties (6). Activated PC might inhibit leukocyte adhesion and transmigration, downregulating endothelial expression of intercellular adhesion molecule-1 (ICAM1) and vascular cell adhesion molecule-1 (VCAM1) (7, 8). In MS-related vascular changes, circulating soluble (s) forms of cell adhesion molecules (CAMs) can result from activated membranes shedding in response to endothelial damage (9).

Neurodegenerative outcomes in MS patients have been also associated with higher plasma levels of C-C motif ligand 18 (CCL18), a chemokine involved in immune cell chemotaxis (10). To note, inflammatory cytokines favor the expression of plasminogen activator inhibitors-1 (PAI1) (11), the key fibrinolysis inhibitor, which counteracts the dissolution of the fibrin clot and may contribute to perturbed fibrinolysis in MS cerebral tissue (12, 13). Interestingly, significantly higher levels of CCL18 and of PAI1 have been reported in MS patients (10, 14).

Among the main regulators of hemostasis, the disintegrin-like and metalloprotease with thrombospondin type 1 motif 13 (ADAMTS13) has been suggested to support vascular integrity (14–16), and ADAMTS13 function has been reported to be also affected by inflammatory profiles (15, 17).

The progressive failure of BBB integrity might have the pathological features of cerebral microbleeds (CMBs) (18), revealed by MRI analysis and associated with worsening of physical and cognitive disability in MS (19). Lower plasma levels of ADAMTS13 in MS and particularly in those with CMBs (14) together with higher levels of soluble vascular adhesion protein 1 (sVAP1) (20) have been reported.

Taking advantage of main findings reported in our previous studies, focused on different biological pathways and performed on the same MS cohort, we hypothesized that circulating concentration of hemostasis inhibitors could participate in the immunity and inflammation MS-related network. To assess this hypothesis, in the current study, (i) we evaluated the combined contribution of the main coagulation inhibitor PC and the CCL18 chemokine levels to MS brain atrophy, (ii) we compared PC and CCL18 plasma concentrations for their ability to explain the observed neurodegeneration, and (iii) we investigated the correlations among circulating levels of hemostasis inhibitors (PC, ADAMTS13, and PAI1), CCL18, and adhesion molecules [sICAM, soluble neural CAM (sNCAM), sVAP1, and sVCAM1], particularly in relation to CMBs.

## Methods

### Study Population

The population used for this analysis included 138 MS patients and 42 healthy individuals (HI) derived from the CEG-MS study. Details on the collection, diagnosis, and demographics of this cohort have been previously described (14). The study protocols were approved by the institutional review boards of the University at Buffalo (USA) (ID:MODCR00000352) and the University/Hospital of Ferrara (Italy) (ID:170585). All participants provided informed consent.

### Plasma Assay

Multiplex magnetic-bead technology (Luminex R&D Systems Inc., Minneapolis, MN, USA; Merck Millipore, Darmstadt, Germany) was used to measure the following panel of hemostasis inhibitors, chemokines, and adhesion molecules: ADAMTS13, PC, PAI1, CCL18, sICAM1, sNCAM, sVAP1, and sVCAM1 (10, 14, 20, 21).

### Magnetic Resonance Imaging Acquisition and Image Analysis

Brain MRI was performed by 3-T GE Signa Excite HD 12.0 scanner (Milwaukee, WI, USA) with an eight-channel head and neck coil. Details of the acquisition protocol and MRI analyses were previously provided (5, 14) and here reported.

Acquisition of two-dimensional (2D) T2/PD-weighted images (WI), fluid-attenuated inversion recovery (FLAIR), spin-echo T1-WI with and without gadolinium contrast, and a three-dimensional (3D) high-resolution T1-WI was performed. 2D sequences were collected using a 256 × 192 matrix and 256 × 192 mm^2^ field of view (FOV), resulting in a nominal in-plane resolution of 1 × 1 mm^2^. For the whole-brain coverage, 48 gap-less 3-mm-thick slices were acquired. The sequence-specific parameters were as follows: dual fast spin echo (FSE) proton density and T2-WI (TE1/TE2/TR = 9/98/5,300 ms; echo-train length = 14), 4:31 min long; FLAIR (TE/TI/TR = 120/2,100/8,500 ms; flip angle = 90°; echo-train length = 24), 4:16 min long; and spin-echo T1-WI (TE/TR = 16/600 ms), 4:07 min long. Last, a 3D high-resolution T1-WI fast spoiled gradient echo sequence with a magnetization-prepared inversion recovery pulse was obtained (TE/TI/TR = 2.8/900/5.9 ms, flip angle = 10°), 4:39 min long, with 184 slices of 1 mm.

For the image analysis, a semi-automated edge detection thresholding technique was used to assess T2- and T1-LV, as previously reported (22). Prior to tissue segmentation, lesion filling was utilized to minimize the impact of T1 hypointensities. SIENAX software (version 2.6) was used to calculate normalized volumes of whole brain (WBV), gray matter (GMV), white matter (WMV), cortex (CV), and lateral ventricles (LVV). Deep GMV (DGMV) and thalamic volume were calculated using FIRST (23) and subsequently normalized using the SIENAX-derived scaling factor (24).

The CMB analysis was performed on susceptibility-weighted imaging (SWI) minimum-intensity projection images and susceptibility maps. CMBs were classified as focal, small, and round to ovoid punctuate areas of signal hypointensity on SWI minimum-intensity projection images, as previously reported (19). Signal voids caused by sulcal vessels, calcifications, and signal averaging from bone were considered mimics of microbleeds. The presence and number of definite CMBs were determined on SWI minimum-intensity projection images by using the Microbleed Anatomic Rating Scale (25). The CMB volume was calculated on susceptibility maps by using a semiautomated edge detection contouring and thresholding technique (22).

### Statistical Analyses

Analyses were performed using SPSS (version 24, IBM, Armonk, NY, USA). Demographic and clinical variables were compared using χ^2^, Student's *t*-test, or Mann–Whitney U-test.

To evaluate the contribution of PC and CCL18 to MS brain atrophy, multiple regression analysis was conducted with each MRI characteristic used as the dependent variable while age, sex, body mass index (BMI), and the plasma protein levels were predictor variables. The first block included the forced entry of age, sex, and BMI; and the second block included the stepwise entry of PC and CCL18 natural logarithmic values. To further determine the findings' validity, multivariate regression analysis by enter method with 1000-sample bootstrapping procedure was performed.

Association analysis of logarithmic values of protein levels in MS patients and HI was performed by partial correlation with 1000-sample bootstrapping procedure, using age and sex as covariates. Due to the low number of MS patients with CMBs, Pearson's correlation with 1000-sample bootstrapping procedure was used to assess associations among logarithmic values of protein levels.

All reported *p*-values are based on two-tailed statistical tests, with a significance level of 0.05.

## Results

### Demographic, Clinical, and MRI Characteristics

Table 1 summarizes the demographic and clinical characteristics of the study population. There were no significant differences in any demographic characteristics between the MS and HI groups.

**Table 1:** Demographic and clinical characteristics of the cohort.

	***N***	**Female (%)**	**Age, year**	**Disease duration, year**	**EDSS**	**Annual relapse rate**
All MS	138	100 (72.5)	54.3 ± 10.8	21.1 ± 10.6	3.5 (2.0–6.0)	0.2 ± 0.4
RR-MS	85	60 (70.6)	50.1 ± 10.7	17.0 ± 8.8	2.0 (1.5–3.5)	0.2 ± 0.4
P-MS	53	40 (75.5)	60.9 ± 7.2	27.6 ± 10.0	6.0 (4.0–6.5)	0.1 ± 0.3
MS with CMBs	12	6 (50)	60.8 ± 8.8	25 ± 11.3	4.0 (3.5–6)	0.1 ± 0.1
HI	42	31 (73.8)	51.0 ± 14.3	n.a.	n.a.	n.a.
MS vs. HI *p*-value		0.99	0.11	–	–	–
RR-MS vs P-MS *p*-value		0.56	<0.001	<0.001	<0.001	0.002

*All continuous variables are shown as mean ± standard deviation. The ordinal EDSS is shown as median (interquartile range)*.

*MS, multiple sclerosis; RR-MS, relapsing-remitting multiple sclerosis; P-MS, progressive multiple sclerosis; CMBs, cerebral microbleeds; HI, healthy individuals; N, number; EDSS, Expanded Disability Status Scale; n.a, not applicable*.

The progressive (P)-MS group comprised 46 secondary-progressive MS patients and seven primary-progressive MS patients. As expected, P-MS patients were older than relapsing-remitting (RR)-MS patients (*p* < 0.001), and the RR-MS and P-MS groups differed in clinical characteristics and brain MRI measures (Table 2).

**Table 2:** MRI characteristics of the study population.

	**T2-LV**	**T1-LV**	**WBV**	**WMV**	**GMV**	**CV**	**LVV**	**DGMV**	**Thalamus volume**
All MS	15.8 ± 19.0	2.9 ± 6.2	1,438 ± 92.1	710.4 ± 44.5	727.6 ± 61.1	591 ± 48.6	55.1 ± 27.0	53.6 ± 7.1	17.7 ± 2.5
RR-MS	11.8 ± 15.9	2.0 ± 4.6	1,469 ± 82.4	721.8 ± 41	747 ± 56.9	606 ± 44.8	50.7 ± 25.2	55.5 ± 6.5	18.4 ± 2.3
P-MS	22.2 ± 21.9	4.4 ± 8.1	1,387 ± 85.2	691.5 ± 44.1	695.8 ± 54.4	567 ± 44.8	62.3 ± 28.5	50.4 ± 6.9	16.5 ± 2.4
RR-MS vs. P-MS *p*-value	0.016	0.075	0.001	0.001	0.018	0.028	0.23	0.007	0.008

*Lesion and brain volumes are expressed in milliliters and reported as mean values ± standard deviation. ANCOVA with age and sex as covariates was used for comparison of MRI volumes*.

*MS, multiple sclerosis; RR-MS, relapsing-remitting multiple sclerosis; P-MS, progressive multiple sclerosis; LV, lesion volume; WBV, whole brain volume; WMV, white matter volume; GMV, gray matter volume; CV, cortical volume; LVV, lateral ventricular volume; DGMV, deep gray matter volume*.

### Measures of Protein Levels and Neurodegeneration: An Integrated Model

To evaluate the combined contribution on MRI characteristics of PC and CCL18 levels, previously found to be associated with neurodegeneration in two separate investigations on the same MS patient cohort (5, 10), integrated regression analyses were performed.

To normalize data, regression analyses were conducted using natural logarithmic values of PC and CCL18. In the whole MS population, higher PC levels were associated with lower GMV, CV, DGMV, and thalamic volume (Table 3). PC alone was able to predict 37, 36, 22, and 25% of total variation of GMV, CV, DGMV, and thalamic volume, respectively. In this model, one logarithmic unit (~2.7 ng/ml) increase in PC was associated with decrease in GMV (40.9 ml), in CV (28.9 ml), in DGMV (4.79 ml), and in thalamic volume (1.7 ml). All associations were confirmed by bootstrap analysis (Table 3).

**Table 3:** Association of PC and CCL18 concentrations with MRI characteristics in multiple sclerosis patients.

	**All MS (*****n*** **=** **138)**						**RR-MS (*****n*** **=** **85)**						**P-MS (*****n*** **=** **53)**					
	**PC**		**CCL18**				**PC**		**CCL18**				**PC**		**CCL18**			
	***r*_**p**_**	***P***	***r*_**p**_**	***p***	***R*^**2**^**	**β [CI 95%] *p*^**#**^**	***r*_**p**_**	***p***	***r*_**p**_**	***p***	***R*^**2**^**	**β [CI 95%] *p*^**#**^**	***r*_**p**_**	***p***	***r*_**p**_**	***p***	***R*^**2**^**	**β [CI 95%] *p*#**
T2-LV	/	0.241	**0.19**	**0.030**	0.106	[0.5, 17.0] 0.057	/	0.088	/	0.952			/	0.802	**0.43**	**0.003**	0.209	[10.7, 39.4] 0.003
T1-LV	/	0.421	/	0.214			/	0.313	/	0.357			/	0.522	**0.34**	**0.021**	0.182	[1.7, 1.6] 0.089
WBV	**−0.27**	**0.002**	/	0.353	0.316	[−90.4, −15.2] 0.006	**−0.31**	**0.006**	/	0.803	0.293	[−94.6, −11.7] 0.013	/	0.274	/	0.112		
WMV	/	0.166	/	0.436			/	0.224	/	0.845			/	0.645	/	0.747		
GMV	**−0.32**	** <0.001**	/	0.279	0.374	[−63.0, −14.8] 0.006	**−0.36**	**0.001**	/	0.760	0.367	[−66.8, −8.0] 0.005	/	0.380	**−0.33**	**0.024**	0.200	[−78.0, −2.8] 0.021
CV	**−0.28**	**0.001**	/	0.217	0.362	[−48.3, −10.8] 0.002	**−0.34**	**0.003**	/	0.922	0.380	[−49.7, −6] 0.009	/	0.606	**−0.30**	**0.043**	0.179	[−60.3, −2.3] 0.027
LVV	/	0.381	/	0.105			/	0.226	/	0.430			/	0.758	/	0.109	/	
DGMV	**−0.26**	**0.001**	/	0.052	0.219	[−7.6, −1.9] 0.003	**−0.35**	**0.002**	/	0.434	0.246	[−8.3, −1.1] 0.008	/	0.452	**−0.32**	**0.029**	0.127	[−10.9, −0.03] 0.042
Thalamic volume	**−0.29**	**0.001**	/	0.088	0.248	[−2.7, −0.6] 0.003	**−0.36**	**0.002**	/	0.724	0.269	[−2.9, −0.4] 0.006	/	0.486	**−0.34**	**0.022**	0.152	[−4.0, −0.3] 0.039

*Regression model: the first block included the forced entry of age, sex, and BMI; and the second block included the stepwise entry of PC and CCL18 natural logarithmic values*.

*Partial correlation (r_p_) and p-value from the regression analysis are shown. R^2^ of the regression model is reported. Predictor variable excluded from the model (/). The 95% confidence intervals (CIs) of β coefficient and the p-value^#^ from the 1000-sample bootstrapping are reported*.

*LV, lesion volume; WBV, whole brain volume; WMV, white matter volume; GMV, gray matter volume; CV, cortical volume; LVV, lateral ventricular volume; DGMV, deep gray matter volume; MS, Multiple Sclerosis; RR-MS, relapsing-remitting multiple sclerosis; P-MS, progressive multiple sclerosis. Significant results are in bold. No significant results are in grey*.

In the whole MS population, higher CCL18 levels were associated with higher T2-lesion volume (LV). CCL18 alone was able to predict the 10% of total variation of T2-LV, and for one logarithmic unit (~2.7 ng/ml) increase in CCL18 was associated with 8.16-ml increase in T2-LV. This result showed a trend for significance in bootstrap analysis (*p* = 0.057).

Sub-analysis of clinical phenotype groups indicated that PC and CCL18 levels were predictors of variation of GM-related volumes in RR-MS and P-MS patients, respectively (Table 3).

In RR-MS patients, one logarithmic unit (~2.7 ng/ml) increase in PC was associated with decrease in GMV (41.2 ml), in CV (30 ml), in DGMV (5.04 ml), and in thalamic volume (1.7 ml).

In P-MS patients, one logarithmic unit (~2.7 ng/ml) increase in CCL18 was associated with decrease in GMV (44.4 ml), in CV (32.4 ml), in DGMV (5.8 ml), and in thalamic volume (2.1 ml) and with increase in T2-LV (25 ml).

### Protein Level Correlations of Protein C and Chemokine C-C Motif Ligand 18 in Multiple Sclerosis and Healthy Individuals

In MS patients and in HI, PC levels were positively associated with CCL18 levels (Table 4).

**Table 4:** Correlations among protein levels in multiple sclerosis patients and healthy individuals.

		**ADAMTS13**	**PC**	**PAI1**
**All MS (*****n*** **=** **138)**				
CCL18	Rho, [CI 95%]	−0.10, [−0.24, 0.06]	**0.28**, [**0.12, 0.44**]	**0.30**, [**0.15, 0.44**]
	*p*-value	0.24	**0.001**	** <0.001**
sICAM	Rho, [CI 95%]	0.09, [−0.12, 0.28]	0.03, [−0.13, 0.18]	−0.14, [−0.32, 0.03]
	*p*-value	0.286	0.77	0.11
sNCAM	Rho, [CI 95%]	0.09, [−0.09, 0.25]	0.01, [−0.15, 0.18]	−0.03, [−0.17, 0.09]
	*p*-value	0.31	0.884	0.700
sVAP1	Rho, [CI 95%]	0.07, [−0.11, 0.25]	**0.20, [0.05, 0.36]**	0.06, [−0.11, 0.23]
	*p*-value	0.39	**0.018**	0.45
sVCAM1	Rho, [CI 95%]	0.00, [−0.14, 0.17]	0.06, [−0.12, 0.22]	**0.25, [0.08, 0.38]**
	*p*-value	0.98	0.51	**0.004**
**HI (*****n*** **=** **42)**				
CCL18	Rho, [CI 95%]	0.25, [−0.09, 0.52]	**0.42, [0.17, 0.64]**	**0.33, [−0.03, 0.66]**
	*p*-value	0.13	**0.008**	**0.045**
sICAM	Rho, [CI 95%]	0.19, [−0.14, 0.41]	**0.33, [−0.04, 0.66]**	0.30, [0.00, 0.58]
	*p*-value	0.26	**0.040**	0.066
sNCAM	Rho, [CI 95%]	0.21, [−0.11, 0.48]	**0.35, [0.02, 0.60]**	−0.04, [−0.30, 0.24]
	*p*-value	0.22	**0.031**	0.83
sVAP1	Rho, [CI 95%]	0.26, [−0.10, 0.50]	0.23, [−0.11, 0.56]	0.27, [−0.09, 0.52]
	*p*-value	0.12	0.161	0.11
sVCAM1	Rho, [CI 95%]	0.20, [−0.07, 0.45]	0.27, [0.03, 0.48]	0.21, [−0.17, 0.52]
	*p*-value	0.22	0.097	0.21

*Significant correlations are in bold. Correlations present only in MS patients are reported in dark gray cells. Correlations present only in healthy individuals are reported in light gray cells*.

*ADAMTS13, a disintegrin-like and metalloprotease with thrombospondin type 1 motif 13; MS, multiple sclerosis; HI, healthy individuals; PC, protein C; PAI1, plasminogen activator inhibitor 1; CCL5, C-C motif ligand 5; CCL18, C-C motif ligand 18; sICAM1, soluble intercellular adhesion molecule; sNCAM, soluble neural cell adhesion molecule; sVAP1, soluble vascular adhesion protein-1; sVCAM1, soluble vascular cell adhesion molecule 1*.

A focused sub-analysis in clinical phenotype groups showed that PC-CCL18 levels were correlated in RR-MS patients (rho = 0.29, *p* = 0.008, CI 95% = 0.08, 0.48) but not in progressive patients (rho = 0.23, *p* = 0.10, CI 95% = −0.04, 0.48).

In both MS patients and HI, CCL18 levels were also positively associated with PAI-1.

PC was associated with sVAP1 only in MS patients, and with sICAM1 and sNCAM only in HI.

### Protein Level Correlations in Patients With Cerebral Microbleeds

In MS patients with CMBs, the correlation between CCL18 and PAI1 (*r* = 0.85, *p* = 0.001, CI 95% = 0.74, 0.97) was even stronger than in MS patients without CMBs (*r* = 0.26, *p* = 0.003, CI 95% = 0.10, 0.42). Similarly, in MS with CMBs, the correlation between PAI1 and sVCAM1 (*r* = 0.64, *p* = 0.026, CI 95% = 0.29, 0.94) was better than in MS patients without CMBs (*r* = 0.21, *p* = 0.022, CI 95% = 0.03, 0.36).

Levels of ADAMTS13 were correlated with those of sVAP1 (*r* = 0.78, *p* = 0.003, CI 95% = 0.42, 0.96). Correlation between ADAMTS13 and sVAP1 was detectable neither/nor in MS without CMBs (*r* = 0.16, *p* = 0.86, CI 95% = −0.16, 0.20) nor in HI (*r* = 0.26, *p* = 0.12, CI 95% = −0.10, 0.50).

**Figure 1:**
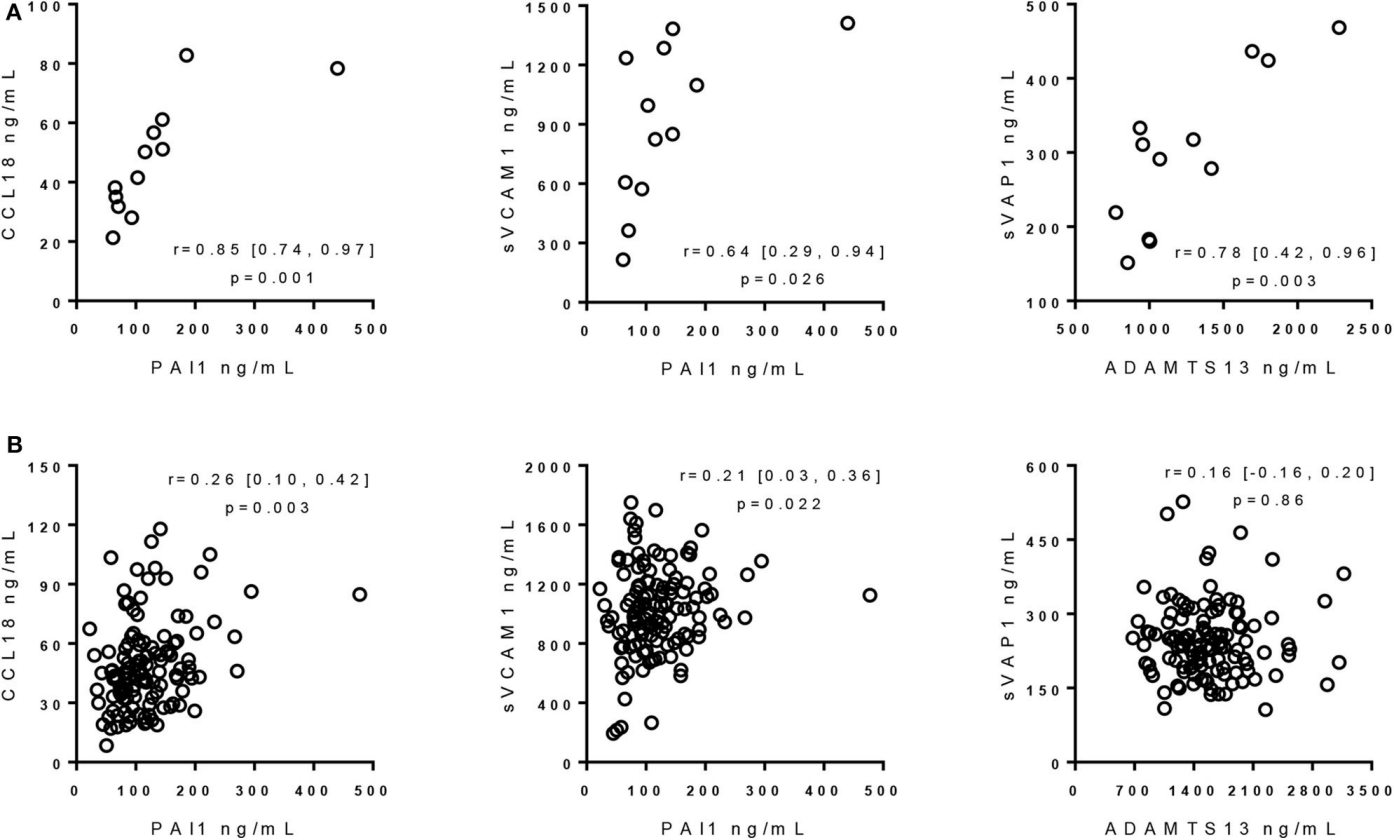
Correlation of protein concentrations in multiple sclerosis patients with and without cerebral microbleeds. **(A)** Correlation of protein concentrations in MS patients with CMBs. Rho, 95% confidence intervals, and *p*-values from Pearson's correlation with 1000-sample bootstrapping procedure, using logarithmic values of protein levels, are reported. **(B)** Correlation of protein concentrations in MS patients without CMBs. Rho, 95% confidence intervals, and *p*-values from partial correlation with 1000-sample bootstrapping procedure with age and sex as covariates, using logarithmic values of protein levels, are reported. ADAMTS13, a disintegrin-like and metalloprotease with thrombospondin type 1 motif 13; sVAP1, soluble vascular adhesion protein-1; PAI1, plasminogen activator inhibitor 1; CCL18, C-C motif ligand 18; sVCAM1, soluble vascular cell adhesion molecule 1.

Scatter plots of protein concentrations in MS patients with and without CMBs are shown in Figure 1.

## Discussion

Our study was aimed at providing an integrated analysis of plasma levels of hemostasis inhibitors, chemokines, and adhesion molecules, found associated with MRI findings in an MS cohort.

The main results of this study were (i) the differences between PC and CCL18 levels in the ability to predict neurodegeneration in MS patients and (ii) the positive correlation between PC and CCL18 levels present in diseased and healthy conditions. Both observations support the hypothesis of a relation between the hemostasis and chemokine pathways, which might act in the disease pathophysiology. Our findings in patients are coherent with previous data: (i) PC and CCL18 levels have been positively associated with neurodegeneration (5, 10), (ii) the proteomic profiles within chronic active plaques have detected the presence of the PC inhibitor (26), which binds activated PC, and (iii) high CCL18 gene expression has been found in the rim of chronic active MS lesions (27).

The different results of the regression analysis between the clinical phenotype groups suggest that the circulating levels of PC and CCL18 might be related to different aspects of neurodegeneration. The relation of PC levels with GM-related volumes in whole MS population and RR-MS could be interpreted as an increase of PC expression-associated with inflammation. This might represent the response to diffuse neuronal loss associated with inflammatory and oxidative injuries, which might occur independently of focal lesions (28). Our data suggest that in patients with slightly higher PC concentration, which did not differ from that in HI, the well-known protective effects of this protein (29, 30) are not sufficient to counterbalance the ongoing neurodegeneration. Differently, the association of CCL18 with T2-LV in the whole MS population, and in particular in P-MS, would be mainly involved in the neurodegeneration mediated by focal lesions. Moreover, the association of this cytokine with GM volume loss in P-MS might be explained by both secondary antegrade (Wallerian) and retrograde neurodegeneration (31).

These hypotheses are strengthened by the analysis of level correlation among proteins related to MRI findings in MS patients, which pointed out the noticeable correlation between levels of PC and CCL18, both associated with neurodegeneration. This correlation would suggest factors able to upregulate expression of both PC and CCL18 by mechanisms that are only partially known (32–35). On the other hand, it has been shown that age, sex, BMI, low-density lipoprotein, high-density lipoprotein, and triglycerides can differentially influence concentration and activity of PC (36–38). A limitation of our study is that the plasma sampling conditions prevented the evaluation of PC activity. However, we expect that higher total PC levels measured in our study are proportional to higher PC activity levels. We can only speculate that mechanism underlying neurodegeneration can affect both PC and CCL18 levels with partially different pathological consequences. This novel hemostasis inhibitors–immunity link is further supported by the positive correlation between PAI1 and CCL18 levels, previously found higher in MS patients than HI (10, 14). As a matter of fact, increased CCL18 gene expression has been found in the rim of chronic active MS lesions (27), and increased PAI1 protein in MS lesions has been associated with impaired fibrin clearance (12, 13), which would contribute to the chronic inflammation [reviewed in (4)].

The hemostasis inhibitors–immunity link was even stronger in the low number of patients with CMBs, who displayed high correlation between CCL18 and PAI1 levels. The high association of PAI1 with sVCAM1 extends this molecular relationship to adhesion molecules. Several differences between MS patients and HI in PC correlation with sICAM1, sNCAM, and sVAP1 concentrations support dysregulation, associated with the MS disease, of the adhesion molecules and PC pathways. The absence of correlation between PC and sICAM and sNCAM in MS patients could be reflected in a decreased inhibition of leukocyte adhesion and transmigration (7, 8).

Based on the high correlation between ADAMTS13 with sVAP1 detected only in MS patients with CMBs, and on low ADAMTS13 (14) and high sVAP1 plasma levels (20), previously observed in the same patients, it is intriguing to speculate that the ADAMTS13 function could be correlated to reactive oxygen species (39) produced by VAP1, an inflammatory adhesion molecule endowed with enzymatic properties (40). The contribution of ADAMTS13 to cerebral vascular integrity is supported by finding low ADAMTS13 activity associated with increased risk of dementia (41), ischemic stroke (42), and subarachnoid hemorrhage (43). On the other hand, intracranial hemorrhages and adverse neurological outcome in stroke have been associated with higher activity of VAP1 (44, 45).

In conclusion, with the limitations of a cross-sectional study and the low number of MS patients with CMBs, the integrated analysis of plasma proteins and MRI measures, here reported, provides evidence for new MS disease-relevant relationships among hemostasis, inflammation, and immunity pathways.

## Data Availability Statement

The datasets presented in this article are not readily available because they are restricted to the Buffalo Neuroimaging Analysis Center. Requests to access the datasets should be directed to Robert Zivadinov, rzivadinov@bnac.net.

## Ethics Statement

The studies involving human participants were reviewed and approved by The study protocols were approved by the institutional review boards of the University at Buffalo (USA) (ID:MODCR00000352) and the University/Hospital of Ferrara (Italy) (ID:170585). The patients/participants provided their written informed consent to participate in this study.

## Author Contributions

NZ, RZ, MR, GM, and FB substantially contributed to the concept and design of the study. MB contributed to assay setup. NZ contributed to data acquisition, analysis, and interpretation. DJ, MB, NB, DR, BW-G, and RZ contributed to data acquisition. RZ, DJ, NB, and BW-G contributed to data interpretation, discussion, and manuscript preparation. NZ, GM, and FB wrote the manuscript. All authors critically revised the manuscript for important intellectual content and contributed to the article and approved the submitted version.

## Conflict of Interest

RZ reports personal fees from EMD Serono, Genzyme-Sanofi, Bristol Myers Squibb, and Novartis for speaking and consultant fees, and grants from Genzyme-Sanofi, Novartis, Bristol Myers Squibb, Mapi Pharma, Keystone Heart, Boston Scientific, Protembis, and V-Vawe Medical unrelated to the submitted work. BW-G reports grants and personal fees from Biogen, EMD Serono, Novartis, and Genentech, and personal fees from Mallinckrodt, outside the submitted work. MR reports grants from Otsuka Pharmaceutical Research and Development and grants from National Institute of Neurological Diseases and Stroke, outside the submitted work. The remaining authors declare that the research was conducted in the absence of any commercial or financial relationships that could be construed as a potential conflict of interest.
